# Sex steroids receptors, hypertension, and vascular ageing

**DOI:** 10.1038/s41371-021-00576-7

**Published:** 2021-07-06

**Authors:** Paul J. Connelly, Helen Casey, Augusto C. Montezano, Rhian M. Touyz, Christian Delles

**Affiliations:** Institute of Cardiovascular and Medical Sciences, University of Glasgow, Glasgow, UK

**Keywords:** Hypertension, Risk factors

## Abstract

Sex hormone receptors are expressed throughout the vasculature and play an important role in the modulation of blood pressure in health and disease. The functions of these receptors may be important in the understanding of sexual dimorphism observed in the pathophysiology of both hypertension and vascular ageing. The interconnectivity of these factors can be exemplified in postmenopausal females, who with age and estrogen deprivation, surpass males with regard to hypertension prevalence, despite experiencing significantly less disease burden in their estrogen replete youth. Estrogen and androgen receptors mediate their actions via direct genomic effects or rapid non-genomic signaling, involving a host of mediators. The expression and subtype composition of these receptors changes through the lifespan in response to age, disease and hormonal exposure. These factors may promote sex steroid receptor-mediated alterations to the Renin–Angiotensin–Aldosterone System (RAAS), and increases in oxidative stress and inflammation, thereby contributing to the development of hypertension and vascular injury with age.

## Introduction

Hypertension is the leading modifiable risk factor resulting in cardiovascular disease and mortality worldwide [1]. Ageing is an important mediator in the development of hypertension and contributes significantly to the rising prevalence of this condition [2]. However, the effects of age on blood pressure are not uniform between sexes. Males experience higher rates of hypertension compared with females, until the sixth decade of life, where thereafter this condition is more prevalent in the latter [3]. Therefore, although blood pressure and hypertension rates increase with age, this interaction is not consistent between males and females.

Despite this, there are no sex specific recommendations for treating hypertension in international guidelines [4, 5]. In the landmark Systolic Blood Pressure Intervention Trial (SPRINT), there was no evidence of differences in target or treatment choice between males and females, although this may be a consequence of being underpowered to detect such differences [6–8]. Females only comprised approximately 36% of the SPRINT cohort, which is in keeping with the chronic underrepresentation of this sex in cardiovascular trials. The exclusion of participants under 50 years ensured mostly postmenopausal females would have been recruited and therefore it is uncertain whether pharmacologic management of hypertension should differ premenopausal females compared to age-matched males. Importantly, although antihypertensive medication are generally used more in females, only 44.8% achieve blood pressure control versus 51.1% of treated males [9]. As a consequence, the age, sex, and hormonal status of individuals, which are paramount in the development of hypertension, are too often ignored to the detriment of patient care.

Vascular ageing describes the progressive decline in endothelial function, vascular remodeling, inflammation, and increased arterial stiffness [10]. Processes responsible for this include activation of proinflammatory pathways, oxidative stress, cell senescence, and the instigation of a vascular smooth muscle cell (VSMC) proliferative phenotype. These processes are also present in the development of hypertension and, as a consequence, are closely related and indeed reciprocal in that vascular ageing may propagate the pathophysiology of hypertension and vice versa. Notably, these processes also appear to be modulated by sex.

The means by which sex interacts with blood pressure and vascular ageing are complex and may result from a multitude of hormonal, chromosomal, or even psychosocial factors [11]. Sex steroids, and the receptors through which they act, are emerging as important mediators in the promotion and maintenance of sexual divergence in blood pressure regulation across the lifespan, and the development of vascular injury with age. In this narrative review we evaluate the relationship between estrogen and androgen hormone receptors, hypertension, and vascular ageing.

## Blood pressure, vascular health, and sex steroids across the lifespan

The blood pressure of males and females are equivalent in childhood, however, rapidly rise and exhibit sexual dimorphism during and after puberty, which coincides with the advent of increased sex hormone secretion and function [12]. The influence of estrogen on female blood pressure can be observed during the menstrual cycle, where blood pressure inversely relates to circulating estrogen levels [13]. Increases in blood pressure are more evident in males, at least until later in life, resulting in males having significantly higher blood pressure than age-matched female counterparts [14]. In a meta-analysis of 23 studies including 3476 non-hypertensive participants, 24-h systolic and diastolic blood pressure was 6 and 4 mmHg higher in males than females, respectively [15].

**Fig. 1:**
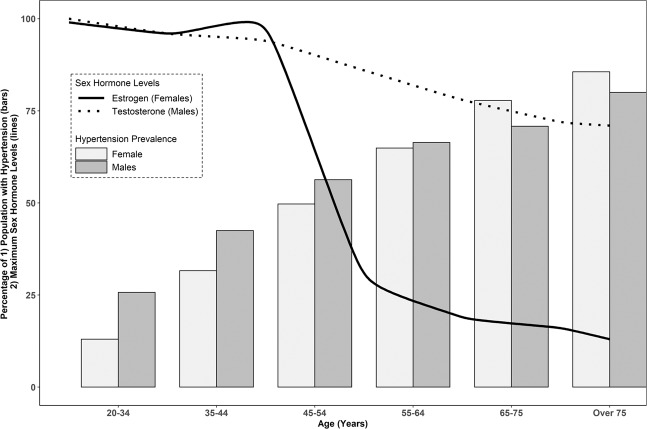
Hypertension prevalence, sex hormone levels and ageing in males and females. Hypertension Prevalence in US Adults between 2013 and 2016 (NHANES) [17]. Sex hormone estimates throughout the lifespan modified from Ober et al. [78].

However, females do exhibit a sharper incline in blood pressure, commencing and persisting from their third decade compared to males [16]. In the United States between 2013 and 2016, the prevalence of hypertension in females and males per age group in the National Health and Nutrition Examination Survey (NHANES) was 13% versus 25.7% (20–34 years), 31.6% versus 42.5% (35–44 years), 49.7% versus 56.3% (45–54 years), and 63.9% versus 66.4% (55–64 years) [17]. Following this timepoint, which would be consistent with the onset of the menopause and loss of estrogen, females consistently demonstrate a higher prevalence of hypertension than males (Fig. 1).

Ageing is associated with reductions in sex hormone levels that may facilitate alterations in blood pressure regulation and promote hypertension and vascular ageing. Testosterone declines by ~1% per year in males over 30 years, while almost a fifth of males over 60 years of age have testosterone levels below normal range values for young males [18, 19]. Although not formally studied in the vasculature, this would be expected to reduce androgen receptor (AR) expression. In a prospective study of males over the age of 50, total testosterone was inversely associated with systolic and diastolic blood pressure and increased risk of death [20]. Similarly, low testosterone is associated with increased pulse wave velocity in older hypogonadal males [21]. Interestingly, this was partially reversed with testosterone supplementation. Data from randomized controlled trials regarding the impact of testosterone on blood pressure are lacking, however, in observational studies of older hypogonadal males, testosterone therapy resulted in decreases in blood pressure [22, 23].

In females, the acceleration in cardiovascular risk and vascular dysfunction following the menopause, and reduction in endogenous estrogen, suggests that the interaction between age and estradiol levels may promote vascular vulnerability. However, in a sub-analysis of the Women’s Health Initiative (WHI) study, conjugated equine estrogen appeared to increase the risk of developing hypertension in older postmenopausal females, which decreased following the discontinuation of this treatment [24]. This effect may be limited to this dose or formulation, as in the Kronos Early Estrogen Prevention Study transdermal or lower doses of conjugated equine estrogen did not alter blood pressure [25].

Taken together, these data suggest that sex steroids are important mediators of blood pressure, however, the effect that these hormones elicit may differ according to the stage of life of an individual.

## Cardiovascular sex steroid receptors

### Estrogen receptors

Sex steroid receptors are expressed throughout the vasculature and sex steroids act through their receptors via genomic and non-genomic mechanisms. Estrogen receptors (ERs) are expressed in endothelial and VSMCs and their actions in these tissues that modulate vascular tone are numeorus [26]. The primary physiological estrogen is 17β-estradiol, which mediates direct genomic signaling, where it binds to cytosolic ER, ERα, and Erβ. These in turn dimerize and translocate to the nucleus where they bind to estrogen response elements (ERE). Alternatively, binding may occur on the activator protein-1 and specificity protein-1, which reside on the promoter of estrogen responsive genes and may modulate transcriptional changes [27]. Estrogen can also bind to membrane-bound ERs (ERα, ERβ, and G protein coupled estrogen receptor), which promote intracellular second messenger signaling, via MAPK/ERK/PI3K/cAMP, that indirectly modulates gene expression and facilitates rapid changes that may alter blood pressure such as increasing NO bioavailability and promoting vasodilatation.

The RAAS plays an important role in the vasculature aberrations that occur with ageing and hypertension and is also modulated by sex hormones [10]. Baseline levels of renin, plasma renin activity and aldosterone are elevated during the luteal phase of the menstrual cycle, where estrogen levels are high compared to the follicular phase [28]. Despite humoral activation of RAAS, mean arterial blood pressure during the luteal phase was not maintained during orthostatic stress, suggesting that estrogen may downregulate tissue responses to RAAS components either through direct ER signaling or through NO-mediated vasodilatation.

ERα appears to be pivotal in the relationship between estrogen and RAAS mediators (Table 1). In the juxtaglomerular cells, ERα directly binds to the ERE in the renin enhancer gene that is required for basal renin expression [29]. Importantly, angiotensin (Ang) II induced hypertension is increased in ovariectomized ERα knockout female mice, compared with intact wild-type [30]. In premenopausal females, an ERα mediated increase in Ang-(1-7) and angiotensin-converting enzyme (ACE) 2 activity promotes a vasodilatory phenotype [31]. The vasodilatory effect of Ang-(1-7) is lost in elderly female mice and restored with estrogen replacement [32]. The combination of ageing and estrogen loss in females may therefore blunt the protective effect of this RAAS component and promote hypertension.

**Table 1:** Estrogen receptor subtype mediated effects associated with hypertension and vascular ageing.

Receptor	Model	Effect
ERα	Murine juxtaglomerular cells	Maintenance of baseline renin expression
	Ang II treated ERα knockout mice	Ang II induced hypertension is increased Increased systolic blood pressure, ventricular weight and vascular contractility Increased renal inflammation and oxidative stress in older mice
	Ovariectomised rats	ERα downregulation and endothelial NO signaling impaired following estrogen deprivation
	Premenopausal human females	Increases in Ang-(1-7) and ACE2 activity
ERβ	ERβ deficient mice	Abnormal VSMC channel function and age associated hypertension
	Spontaneously hypertensive rats	ERβ activation reduces blood pressure
	Human females	ERβ polymorphisms are associated with salt-sensitive blood pressure and hypertension

ERα consists of six domains containing two independent activation functions, AF-1 and AF-2. Models of ERα inactivation (ER−/−) and selective inactivation of nuclear ERα actions, through activating function 2 (AF2^0^) deletions, or membrane-initiated ERα actions via point mutations of the palmitoylable Cys451(C451A), have elucidated pathways by which estrogen elicits its cardioprotective effect [33]. In mice treated with Ang II, increased systolic blood pressure, ventricle weight, and vascular contractility were evident in ERα−/− and AF2^0^ mice compared to either wild-type or C451A mice. Moreover, renal inflammation and oxidative stress were increased in old hypertensive ER−/− and AF2^0^ mice, compared to old hypertensive wild-type and C451A mice. Therefore, nuclear ERα-AF2, and not extra-nuclear ERα signaling, appears to play a protective role in Ang-II dependent hypertension and target organ damage in ageing mice.

There is also evidence for the role of ERβ in the modulation of blood pressure, however, the mechanisms underpinning these actions are less clear (Table 1). ERβ-deficient mice develop abnormalities in VSMC ion channel function and age associated hypertension [34]. Ligand-mediated activation of ERβ also promotes reductions in blood pressure in spontaneously hypertensive rats (SHR) [35]. Interestingly, direct activation of ERβ was found to be more potent than stimulation through the nonselective use of 17-β estradiol. Moreover, in humans polymorphisms in ERβ have been found to be associated with salt-sensitive blood pressure and hypertension [36, 37].

Importantly, ER expression and the balance of ER subtypes can change with ageing and prolonged estrogen deficiency, which in turn alters responses to estrogen [38]. In a small sample of postmortem coronary arteries, VSMC ER expression was lower in postmenopausal versus premenopausal females, while atherosclerosis lowered ER expression independent of menopausal state [39]. Animal studies have demonstrated that endothelial ERα expression is downregulated and endothelial NO signaling is impaired following extended periods of estrogen deprivation [40]. This does not recover following the reintroduction of this sex hormone. In endothelial cells obtained from peripheral veins, ERα expression fluctuated throughout the menstrual cycle in response to estrogen, and was reduced in estrogen-deficient postmenopausal females [41]. Age-related methylation of ER promotor regions, histone deacetylation, and inhibition of membrane localization via posttranslational modifications may mediate senescence-related regulation of ERα expression and function [42]. Conversely, in uterine arteries of postmenopausal females there is a progressive increase in ERβ expression [38]. Consequently, alterations in ER subtype expression with age and declining estrogen levels, may mediate a shift in ERα: ERβ receptor ratios and promote an adverse vascular phenotype and contribute to the development of hypertension and vascular injury.

### Androgen receptors

Testosterone and its more potent metabolite, dihydrotestosterone (DHT), are ligands of the AR. Much like the ER, the AR is expressed in both endothelial and VSMCs. The AR consists of a 110 kDa protein receptor with three major functional regions for transactivation, a DNA binding domain and a hormone binding domain [43]. Two AR variants have been discovered, AR-A and AR-B, with the latter predominating in the most tissues where both receptors are expressed [44]. The exact role of these receptor subtypes has yet to be elucidated.

Unbound cytosolic ARs are co-localized with a number of chaperones, such as heat shock proteins and cytoskeletal elements [44]. After binding, the receptor undergoes a conformational change, resulting in a dissociation of these chaperones and promotes AR dimerization and nuclear translocation, where its interactions with androgen response elements (ARE) to modulate genomic responses [45]. Alternatively, non-genomic testosterone effects are mediated by membrane-bound ARs and act via multiple pathways including PKA, PKC, and MAPK [46]. Through these non-genomic pathways, testosterone can stimulate rapid vasodilatation via endothelium dependent and independent mechanisms [47]. The former result from increased NO bioavailability via AR-mediated eNOS activation, and the release of vasodilating factors into the VSMCs.

Testosterone increases renin levels and expression/activity of ACE and AT1R, while downregulating AT2R, thereby favouring a vasoconstrictor pathway [48]. In models of hypertension, such as in New Zealand genetically hypertensive rats, androgens enhance vascular responsiveness to Ang II [49]. The interactions between these factors may therefore be important in the development of hypertension. Indeed, Ang II induced vascular contraction appears dependent on androgen status. Chronic testosterone deficiency via castration ameliorates Ang II induced increases in blood pressure [50]. Conversely, the hypertensive effect of Ang II is exaggerated in males with intact testis and castrated males that received exogenous testosterone replacement. Therefore, testosterone may modulate the development and maintenance of Ang II induced hypertension and increased vascular contractility to pressors. Interestingly, testosterone supplements in young non-hypogonadal male SHR resulted in increases in blood pressure, which is mediated via RAAS. However, when administered to older SHRs a decrease in blood pressure was observed [51]. Consequently, both the testosterone status, which may alter AR expression, and the age of recipient may influence the blood pressure response to this hormone.

### Molecular mechanisms of sex steroid mediated hypertension and vascular ageing

In addition to direct effects of sex hormones on vasodilation via the NO system, or indirectly via the RAAS, sex steroids may modulate a number of mechanisms evident in the development of both hypertension and vascular ageing.

## Oxidative stress

Oxidative stress plays a central role in the development of vascular ageing in hypertension. In rat coronary arterioles, both age and loss of circulating estrogens, as a consequence of ovariectomy, reduce NO bioavailability. Importantly, dilatation of these arterioles are highly dependent on this mechanism. Impairment in reactive oxygen species (ROS) regulation, especially O_2_-, appears to modulate this decrease in NO-mediated dilation as a consequence of age or estrogen deficiency [52]. Decreases in Cu/Zn superoxide dismutase expression in both aged and ovariectomized rats were observed. This was restored following estrogen replacement in young rats. This phenomenon has also been observed in mice, where the aortic contractile response to thromboxane A2 is modulated by the interaction between NO synthase (NOS) and cyclooxygenase (COX) pathways [53]. This interaction is dependent again on age and estrogen status and promoted by COX-mediated generation of superoxide that decreases NO bioavailability. Of note, increases in the ERβ: ERα ratio that appear to occur with ageing are associated with increased oxidative stress [54].

The relationship between testosterone and redox status is also complex. The AR modulates increased expression of a number of pro-oxidant enzymes such as NAD(P)H oxidases, xanthine oxidases and COX-2. Furthermore, AR may increase the transcription of genes related to the c-Src and PI3K/Akt pathways, which also promote ROS generation [55]. In a rat model of Ang II induced hypertension, ROS generation was increased by testosterone, only in hypertensives through phosphorylation of c-Src, an upstream regulator of NADPH oxidase [56]. However, these effects appear to be determined by testosterone status and ageing. In a rat model of ageing and testosterone deprivation, a decrease in antioxidant haeme oxygenase activity was observed and reversed with testosterone supplementation [57]. Moreover, testosterone also induces AR-mediated mitochondrial-associated ROS generation and apoptosis in VSMCs [58]. Orchiectomized male rats receiving testosterone replacement demonstrated an improved cardiovascular redox state, thereby reversing elevations in lipid peroxidation and nitrotyrosine [59]. In line with this, low testosterone levels are associated with enhanced oxidative stress and in males with type 2 diabetes and a mean age of over 50 years [60], however, it is unclear whether testosterone supplementation is capable of restoring this balance.

## Inflammation

Inflammation plays a significant role in both the pathogenesis of hypertension and cardiovascular ageing. Via the ERs, estrogen has demonstrated the capacity to reduce the inflammatory response by negatively modulating proinflammatory mediator expression, which likely contributes to the cardioprotective role of this sex hormone [61]. Following balloon injury of the right carotid artery of ovariectomized rats, estradiol significantly reduced the expression of adhesion modules (P-selectin, vascular cell adhesion molecule-1 (VCAM-1), and intercellular adhesion molecule-1 (ICAM-1)), chemoattractants (cytokine-induced neutrophil chemoattractant 2β (CINC-2β), monocyte chemoattractant protein 1 (MCP-1)), and proinflammatory cytokines (IL-1 and IL-6).

However, these effects may also be age-dependent. In murine VSMCs and bone marrow-derived macrophages, the estrogen-mediated reduction in inflammatory response to C-reactive protein occurred only in female cells derived from young and not aged mice [62]. Similarly, in uterine arteries of postmenopausal females, ageing was associated with a switch from an anti-inflammatory to proinflammatory profile [38]. In particular, an increased correlation was observed MCP-1 and the adhesion molecules soluble VCAM-1 and ICAM-1 with ageing. These data further corroborate the hypothesis of the influence of altered ER subtype ratio promoting an adverse vascular phenotype, as increased ERβ expression with ageing was observed.

The immunomodulatory role of androgens, and in particular testosterone, has long been theorized due to the greater incidence of immune-mediated diseases in females and androgen deficient males [63]. Evidence exploring the relationship of between testosterone, hypertension and vascular ageing is limited, however, inference from other vascular models pertains to the immunomodulatory role of the AR in this context. In a murine inflammatory abdominal aortic aneurysm (AAA) model, castration promoted AAA formation via the expansion of inflammatory macrophages and IL-6 and IL-1β upregulation [64]. Conversely, following the administration of testosterone, AAA formation was found to be inhibited by the amelioration of macrophage-mediated inflammatory responses. In human endothelial cells the AR mediates the downregulation of adhesion molecules, chemokines and proteases induced by TNFα, following exposure to DHT [65].

It has been recently demonstrated that testosterone may provide a protective effect on vascular ageing by improving vascular remodeling through the Growth arrest-specific protein 6/Axl pathway, which has been implicated in cell survival, adhesion, migration and inflammatory cytokine release [66]. These findings are consistent with the results of a randomized control trial of testosterone replacement in older hypogonadal males, where testosterone was found to reduce TNFα, IL-1β and increase IL-10, thereby promoting an anti-inflammatory state [67].

### Beyond the vasculature

Beyond the evidence provided in this review, sex hormones may play a wider role in blood pressure regulation through central nervous system, renal involvement and effects on cardiac output and temporal peripheral resistance. For instance, ERs are expressed in the subfornical organ, the paraventricular nucleus and the rostral ventral lateral medulla [68]. These are key regions that regulate sympathetic nerve activity (SNA), which when increased has been implicated as a primary precursor of hypertension. Young premenopausal females have been shown to have lower SNA, than do males of the same age, whereas postmenopausal females have resting muscle SNA that is similar to those of age-matched males [69, 70]. In postmenopausal females long term transdermal estrogen administration decreased SNA and was associated with significant reduction in 24-h ambulatory BP [71, 72]. Similarly, ARs are widely expressed in the central nervous system, however, their role, or indeed the influence of fluctuating sex steroid receptor expression, in the central nervous system in modulating SNA is unclear. Ultimately, a multi-system approach will be required to develop a comprehensive understanding of the role of these receptors in the development of hypertension, and how their age-related plasticity may modulate this relationship.

### Sex-dependent hormonal action

Another factor that must be considered is the sex-dependent role of vascular androgen and ERs. For instance, it has been demonstrated that raised free androgen index (i.e. the ratio of circulating testosterone to sex hormone binding globulin) in postmenopausal females is associated with hypertension and vascular ageing [73]. Therefore, although one system of sex steroid receptor may predominate in a particular sex, both are likely to be of physiological significance, particularly if the other is perturbed. However, the definitive role of ARs in females and ERs in males with respect to the development of hypertension and vascular ageing has not yet been ellucidated [47, 74].

This putative interaction may be of particular importance to the long-term vascular health of transgender individuals receiving gender-affirming hormonal therapy. There is currently insufficient data to advise the impact of sex steroids on blood pressure in this population, however, some studies suggest a higher rate of hypertension [75, 76]. It remains unclear to what effect the administration of physiological concentrations of estrogen or testosterone has in natal males and females, respectively, on sex steroid receptor expression or function. This has the added complexity that unless doses of these hormones are adjusted, age-relative supraphysiological levels will be achieved in older individuals, and the long-term vascular sequelae of this is uncertain [77].

## Conclusions

Mechanisms whereby sex steroid receptors mediate shared processes in both hypertension and vascular ageing are becoming evident. A common theme in the evidence provided is that both the sex hormone status of an individual and their physiological age are important determinants of their response to sex steroid administration. Estrogen, despite eliciting a number of cardioprotective effects in females in youth, may facilitate vascular injury later in life following periods of deprivation. The plasticity of receptor subtype expression in ageing estrogen-deficient females appears to be of particular importance in this population. Conversely, the increased cardiovascular risk in males elicited by testosterone rises further through the relative hypogonadism and age, and is potentially rescued through subsequent re-exposure. It is clear that much work is required to understand the mechanisms by which sex steroid receptors modulate the development of hypertension and vascular ageing, and how these relationships can be exploited to prevent such conditions.
